# Telehealth Simulations with Generative Artificial Intelligence in Midwifery Education: Practice for Person‐Centered and Culturally Responsive Care

**DOI:** 10.1111/jmwh.70015

**Published:** 2025-08-20

**Authors:** Hannah Cole McGrew, Kendra Faucett, Regina G. Russell, Jo Ellen Holt, Jannyse Tapp, Julia Steed, Julia Phillippi

**Affiliations:** ^1^ Vanderbilt University School of Nursing Nashville Tennessee

**Keywords:** artificial intelligence, bias, clinical decision‐making, culturally competent care, health equity, midwifery education, nursing education, person‐centered, simulation, telehealth

## Abstract

The International Confederation of Midwives Essential Competencies and the American College of Nurse‐Midwives Core Competencies for Basic Midwifery Practice include essential skills needed for safe entry‐level practice and provision of person‐centered care to individuals from diverse backgrounds. However, opportunities for midwifery students to interact with diverse patient populations may be limited, especially in homogenous areas. Education programs struggle to recruit standardized patients from wide‐ranging social, cultural, ethnic, and religious backgrounds. In addition, midwifery students may lack skills or experience in providing culturally responsive care, potentially affecting patients and exacerbating health disparities. This article reports on the pilot use of an online artificial intelligence (AI) simulation platform to prepare midwifery students for person‐centered telehealth with culturally and socially diverse, underserved patients. The platform used generative technology to produce interactive avatars with detailed histories and allowed spontaneous and adaptive conversations between the virtual patient and midwife‐in‐training. Ease of use, avatar fidelity, ability to incorporate diverse cultural elements, student learning, time, and cost in the development were assessed. Case development requires collaboration and an iterative approach. Similar to traditional simulation, AI‐based simulations require careful planning, pre‐ and debriefing discussions, and continuous improvement efforts for maximal student learning. Generative AI‐based simulations can enable efficient, flexible preparation for patient‐centered, culturally appropriate telehealth.

## BACKGROUND

There is a large and rising demand for certified nurse‐midwives and certified midwives (CNMs/CMs) who can provide person‐centered care sensitive to an individual's culture and preferences.^1^ The International Confederation of Midwives Essential Competencies^2^ and American College of Nurse‐Midwives Core Competencies for Basic Midwifery Practice^3^ stress that health care should be based on evidence and incorporate patient needs and preferences with respect for their culture and context. In addition, midwives must be prepared to use technology, such as telehealth, to connect with patients and improve access to health care.^3^, ^4^ This is especially relevant for midwives working with rural and underserved populations, who experience social, transportation‐related, and economic barriers to care.^4^, ^5^, ^6^

Although midwives must be prepared to provide person‐centered care appropriate to the person's culture and needs, clinical experiences are often limited to individuals within accessible settings.^1^ Simulation‐based learning has been shown to increase knowledge of cultural diversity, empathy, and cross‐cultural communication, as well as the ability to ask culturally relevant questions and address unique health needs.^7^ In addition, simulation provides an opportunity to develop skills in cultural responsiveness and humility in a nonjudgmental, academic environment without compromising patient safety or negatively impacting the patient‐provider relationship. Artificial intelligence (AI)‐driven simulations can expose midwifery students to a wide range of complex patient scenarios, including those they may not encounter during traditional clinical rotations. Such comprehensive training equips future midwives to address the unique needs of diverse populations, thereby contributing to providing more equitable health care, particularly in rural and underserved communities.^7^

  

Continuing education (CE) is available for this article. To obtain CE online, please visit http://www.jmwhce.org. A CE form that includes the test questions is available in the print edition of this issue.

Traditional clinical simulations allow learners to interact with human patient actors to mimic patient‐provider interactions, permitting practice within a safe environment.^8^, ^9^ Standardized patients are frequently used in simulations, especially when reproducibility is essential.^10^ The Society of Simulation in Healthcare defines a standardized patient as a person who has been coached to simulate an actual patient so accurately that a skilled clinician cannot detect the simulation.^10^ Similar to clinical care, reliance on local individuals as standardized patients may limit the recruitment of people from diverse backgrounds. An especially daunting challenge in rural health care is limited access to specialized training and resources. Simulation‐based education, including AI‐powered tools, can mitigate this by offering realistic, immersive simulations that replicate complex clinical situations and can be accessed from anywhere with broadband internet access.^1^ AI‐based simulated experiences can foster cultural humility by providing learners with opportunities to engage with virtual patient avatars from diverse backgrounds, preparing them to provide high‐quality, culturally competent care across different patient demographics.^11^, ^12^

**QUICK POINTS**

✦ Midwifery students need opportunities to practice and improve their provision of person‐centered and culturally responsive care without adversely affecting patient care.
✦ Computer platforms using generative artificial intelligence (AI) are a feasible and sustainable method for student practice of culturally appropriate and evidence‐based health care.
✦ Simulation is an effective teaching technique for which generative AI can be leveraged to provide immersive learning experiences.

Simulations with human standardized patients can be implemented in very realistic environments, known as high‐fidelity simulations, and have demonstrated impact on the quality and safety of patient care.^12^ High‐fidelity simulations require dedicated student and faculty time, must be completed synchronously, and pose resource and logistical challenges. Although well‐trained standardized patients can ensure replicability across one cohort of simulation, it can be difficult to recruit the same individuals over multiple cohorts. In addition, the number of standardized patients available affects the time needed for all learners to complete the activity, although students can learn through acting as the clinician, and observer, or a colleague, allowing many students to participate in each simulation run.^13^ Substantial time is needed to recruit standardized patients, coordinate experiences, and arrange payment. If a standardized patient cancels or the simulation is rescheduled productivity can be lost for faculty and students.

Generative AI may be useful in simulation for midwifery and other health professions students as avatars can provide unscripted responses to student interactions within a safe learning environment.^8^ It is also possible for the avatars to have detailed medical needs, preferences, and social histories to help students grow their ability to provide patient‐centered, culturally responsive care. Online platforms do not require students to be in one location and can accommodate multiple learning sessions simultaneously, decreasing the overall time needed within the curriculum as well as physical simulation space. Students can receive customized, rubric‐based feedback on their performance without the need for direct or continuous supervision from faculty. In addition, students may repeat the simulation multiple times to gain knowledge and skills.

Within online simulations, patients can include humans interacting in real time or computer‐generated avatars. Although avatars decrease the need for trained actors, these platforms have, until recently, been limited to scripted interactions. Computer software using generative AI has been trained on large amounts of sound, image, or text‐based data and then generates spontaneous responses. AI‐driven simulations offer potential to advance midwifery education by exposing students to realistic clinical scenarios and health challenges faced by rural and underserved populations, while remaining cost‐effective and learner accessible.^14^ By leveraging these innovations, educators can better prepare midwives to practice in a wide range of communities.

AI‐based simulation use in midwifery is not without concerns. Although the use of AI in education has a high level of acceptability with students, midwives have traditionally been skeptical of AI.^15^ The quality of generative AI is based on the quality of the data that it is trained on.^16^, ^17^ Implicit bias in research and information about care provision used to train AI may create poor quality data inputs for marginalized and understudied populations.^16^, ^17^ Ongoing work is examining ways to identify and correct biases in AI models.^16^, ^18^, ^19^ Additionally, generative AI is known to have unpredictable responses referred to as hallucinations, however these can occur with human actors as well.^20^

The goal of this simulation was to prepare midwifery students for person‐centered telehealth with culturally diverse, underserved patients. Cases were developed with input from faculty members from different disciplines and personal social backgrounds. We used a generative AI‐based platform, Re:course AI, that allowed input of patient characteristics for a patient avatar and a rubric for providing student feedback using a quantitative score and a customized written assessment. This article describes the development and implementation of this pilot. Faculty plan to further evaluate these simulations following completion of additional cohorts of students.

## SIMULATION USING GENERATIVE ARTIFICAL INTELLIGENCE

### Development

Using the National League for Nursing Jeffries simulation framework as a guide,^21^ 2 telehealth scenarios with detailed patient social histories and health record information relevant to the presenting concern of amenorrhea were developed by midwifery and family nurse practitioner faculty. (See Table 1.)

**Table 1:** Simulated Patient Scenarios

Case Elements	Case A: Amber Luckett	Case B: Ubah Dahir
Presenting concern	She has not had a period in 2 months and that period was just light spotting (amenorrhea).	Missed period (amenorrhea).
Sociocultural background	Patient lives in a rural area. Her home is a mobile home on her grandparents’ property. Her boyfriend is currently out of state for employment. Her sister and her children live on the grandparents’ land and help with childcare. She is a high school student and pleased to be graduating soon.	The clinic is in a medium‐sized US town with a sizable Somali immigrant population. The patient is a first‐generation Somali American who is a practicing Muslim, consumes only halal foods, and uses hijab in public. She is a television news reporter with few job‐related hazards. She is recently married and is surprised to have become pregnant so quickly. She is experiencing normal ambivalence associated with the first trimester of an unplanned but desired pregnancy.
Relevant history from chart review	Amber is a 17‐year‐old gravida 1, para 1, who is 6 months postpartum. After her uncomplicated vaginal birth, she had a postplacental IUD insertion. On hospital discharge, she was breastfeeding.	Ubah is a 25‐year‐old patient at your practice since she established care for well‐woman care after her marriage last year. She has been seen twice since then: once to get tested for COVID and flu, and once for a vaginal yeast infection.
Essential patient history to be obtained	Menstruation history: no period for 2 months, light spotting prior. Home pregnancy test results (multiple) are negative. Currently breastfeeding at night, and the infant receives a store‐brand infant formula while she is away at school or work.	First day of last menstrual period (5 weeks ago) Signs and symptoms of early pregnancy: Cramping without vaginal bleeding. Home pregnancy test result was positive.
Primary diagnosis	Amenorrhea secondary to progesterone‐containing IUD use.	Positive home pregnancy test.
Essential information to provide patient	Typical menstrual patterns associated with progesterone‐releasing IUDs. Warning signs of pregnancy, including ectopic pregnancy.	Calculate due date and estimated gestational age. Anticipatory guidance for first trimester. Early pregnancy and ectopic pregnancy warning signs and safety. Recommend prenatal vitamins that consider halal and dietary needs (no pork in capsule, need for additional supplementation). Interval for follow‐up visit to initiate prenatal care, laboratory tests, and ultrasound.

Abbreviation: IUD, intrauterine device.

For the first case, faculty with experience in provision of rural care collaborated to develop a composite case and patient communication attributes consistent with the composite patient's age, education level, and cultural background. Our initial case, Amber Luckett, was a 17‐year‐old individual living in a rural area in Appalachia. The faculty had experience providing care to adolescent patients in rural Tennessee and Kentucky. The authors of this article have varying racial and religious identities, and all currently reside in the greater metropolitan area of a large, Southern US city. Several have previously resided or were raised in rural or underserved areas both in the United States and abroad.

The second case, Ubah Dahir, was developed by faculty with expertise in providing clinical care to ethnically diverse, underserved populations, specifically first‐ and second‐generation Somali and Muslim patients. The faculty member leading this case development has experienced immigrating to a new country with limited access to health care. Elements of this simulated case were an amalgamation of real patient cases, including cultural preferences for care, encountered by this faculty member in clinical practice.^21^ There were no faculty at this institution with this patient's specific demographics available to consult on this case; however, the case was reviewed and edited by a group of faculty members and beta tested to assess accuracy and individual bias.

The AI simulated patients were programmed in the platform. For our scenarios, the patients presented with medical needs that students had already encountered in coursework and clinical practica to allow students to focus on practicing person‐centered, culturally responsive telehealth services. Patient‐related information was programmed into a platform with an AI large‐language model trained on publicly available reference sources, content authored by experienced clinicians, and input from patient advocates who provided feedback on language, tone, and cultural responsiveness. Because the content was fully deidentified at the source, Health Insurance Portability and Accountability Act restrictions on the use of protected health information for machine‐learning did not apply. A faculty‐designed rubric was also programmed into the platform to assess student performance and provide text‐based feedback and a numeric score. The platform was able to incorporate cultural and socioeconomic components into the patient scenarios. For example, if prompted, Ubah would discuss her vegetarian diet and halal cultural preferences. Within the Amber scenario, the background simulated the inside of a mobile home, and Amber would discuss living on her family's land, eating food from their farm, and working to finish high school. Once the avatars were programmed, authors (HM, JP, RR, KF) beta tested the simulations and provided suggestions to the programming team, who incorporated those edits into the version provided to the students. Students were asked for feedback regarding the case and behaviors of both avatars following the simulation. This work was reviewed and approved by the Vanderbilt University Institutional Review Board (IRB #240550).

### Implementation

Ten midwifery and dual midwifery and family nurse practitioner students participated in the 2 AI simulations. Following 30 minutes of in‐person instruction about telehealth, students were paired to allow the learners to alternate lead and observer roles for the 2 scenarios. Groups began each online telehealth simulation at the same time and were given 20 minutes for completion and 5 minutes for unstructured peer‐observer feedback. Each simulation was divided into 2 parts. During the first part, the clinician student was asked to obtain a complete history of present illness and generate differential diagnoses. Following the history, the clinician student in the pair was expected to present relevant patient education and a potential plan of care to the simulated patient. A second student participated as an observer during each simulation. Observer students were asked to take unstructured notes as needed and be available for up to 2 instances as a colleague consultation for the student in the clinician role. The observers provided formative feedback to their peers on simulation conclusion. The observer role was included as observation is valuable in student learning, can assist anxious or stuck colleagues, and allows for peer‐to‐peer feedback.^13^ Roles were exchanged between simulations.

After simulation completion, the platform provided each group with a paragraph of detailed performance feedback in relation to the rubric along with a quantitative performance score (Appendix S1). All feedback was solely for the student's personal growth and not associated with a formal grade. Students were allowed several minutes to read the feedback, and then faculty conducted a debrief using the Debriefing for Meaningful Learning (DML) model.^22^

The group debrief allowed students to reflect on their actions and learn from peers.^22^ In the DML model, faculty guided the students through a structured debrief using a whiteboard and 4 colors of markers (black, green, red, and blue). The students reported their step‐by‐step clinical interactions with the patient, and the colors were used to identify correct and incorrect actions, as well as actions that were omitted or could be improved.^22^, ^23^ Students also compared how the avatar patient responded based on their questions and reflected on potential effects of their personal biases

### Evaluation of the Simulation

#### Faculty Feedback About the Simulation

The implementation team informally evaluated the development and pilot of these AI‐based cases. Our educators found the avatar conveyed a range of emotions (such as concern, anxiety, and exasperation) via word choice, facial expressions, and body movement. Faculty observed that the avatars interacted differently depending on students’ questions. In the Ubah scenario, the patient desires their pregnancy and is experiencing typical anxiety and ambivalence. However, when one student continued pressing about patient ambivalence, the simulated patient stated she was potentially interested in having an abortion. Although a well‐studied debriefing framework was used,^22^ faculty felt the debriefing model may need modifications to be more relevant to the dialogue‐based scenarios with AI avatars.^22^

#### Student Feedback About the Simulation

Following each simulation debrief, students were asked to provide feedback about: (1) the quality of the prebrief and debrief, (2) assessment of their learning, and (3) expectations for transfer of learning in simulation to practice. Feedback was captured on the REDCap platform and verbally.^24^ In discussion, students appreciated the thorough prebrief and telehealth lecture that preceded the simulations. There were concerns prior to implementation that a difference in interaction with avatars rather than humans would be off‐putting to students. However, the students enjoyed interacting with the avatars and felt the slight delay between their question and the avatar's response allowed them to collect their thoughts.

Like faculty, the students felt the debrief could be more responsive to the unique aspects of this AI modality and unplanned avatar responses. For example, to several students, Amber stated that her best friend's father was her main social support (despite not being programmed to say this), which made students concerned about abuse. The debrief should address the limitations and pitfalls of generative AI, including anomalous behaviors of avatars that deviate from the planned encounter.

#### Cost Assessment

Simulation costs can be divided into development, occurrence‐based, and related factors.^9^ Development of new simulations usually involves at least 8 hours of faculty time as well as time for an expert to review the case for completeness, accuracy, and feasibility.^9^ Generative AI simulations require additional faculty time to work with programmers to build the case within the online platform. Although programming costs vary, a typical range for new case development is $5,000 to $9,000. The generative AI patient platform presents advantages compared with standardized patient modalities. The largest costs for the online platform are the initial buildout and the monthly per‐learner access fees. However, the online platform does not have occurrence‐based costs such as recruiting, training, and employing standardized patient actors.^9^

## DISCUSSION

The AI‐enabled simulations facilitated efficient practice of patient‐centered, culturally appropriate telehealth. The simulations permitted learners to conduct clinic visits while also developing skills in culturally and socially responsive care. The online AI simulation platform offers efficiency advantages over simulations with standardized patients. Multiple students can complete the simulations simultaneously, reducing the time required in the curriculum for on‐site programs. The simulations can also be completed remotely or asynchronously. Asynchronous and remote access is an important element for midwifery education programs when students are remote from the academic hub and have varying clinical schedules. Although this simulation was implemented with students on campus, the simulations and debriefing could be completed remotely.^25^

AI‐driven simulations have the potential to increase health equity by improving learners’ ability to care for rural and underserved populations. By exposing students to varied populations and cultural contexts through safe simulation environments and debriefing their experiences, students can develop critical skills in cultural sensitivity and competence, enabling them to provide appropriate care across diverse communities. Furthermore, understanding and respecting traditional health practices enhances the ability of health professionals to provide care aligned with patients’ cultural beliefs and promotes health equity.^26^ This practice can increase their confidence and improve their clinical decision‐making, ultimately leading to better outcomes.^15^ Practicing providing care across diverse communities is an important consideration because midwives work with a wide range of individuals throughout their careers.

Although the upfront cost of developing the AI simulations is greater than other simulation methods, the cost per student decreases if simulations are used over multiple cohorts or over repeated attempts by the same individuals. Furthermore, the ability to modify and update the simulations to incorporate new clinical scenarios or cultural elements can maximize investment over time.

A survey was used to evaluate students' perception of the experience, including the value of the prebrief and debriefing sessions, and how they anticipate using the concepts learned in the simulation. Student feedback regarding simulations will be obtained across 4 years and approximately 40 students. Although the feedback thus far has been overarchingly positive, our small sample prevents definitive conclusions, and there are still some notable challenges and lessons learned in piloting the use of this technology with learners. The first group of learners did concur with faculty that the approach to debriefing could be improved for future iterations. Debriefing is especially important in AI‐based simulation activities that have a wide range of possible outcomes depending on individual learner choices along the way. A responsive debrief is necessary to capture and explore unscripted outcomes and to ensure that student experiences and AI‐generated feedback aligned with practice standards and evidence‐based care.

Ensuring the cultural authenticity and sensitivity of the simulations will be an ongoing challenge, and continuous improvements will be made to capture diverse perspectives and avoid perpetuating stereotypes or biases via avatars.^27^ Because the inputs for generative AI reflect the biases and pitfalls of the humans that create them, it can be difficult to identify ways that implicit bias may be baked into scenarios. Simulations will require ongoing feedback from a wide range of students, faculty, preceptors, and community members to guide updates. Another limitation is the difficulty of measuring longitudinal outcomes, or transference, of learning into later patient care, especially as it pertains to optimizing health equity.^10^, ^15^ Although measuring the impact on patient care is a challenge across all simulation modalities, it is important to work toward a better understanding of how generative AI in midwifery education ultimately affects the goal of improved patient health and equity outcomes. Future studies are needed to evaluate the transferability of AI‐based simulation in midwifery education on equity of care across diverse populations and clinical settings.

Interprofessional education was identified by faculty as an area in which simulations could also be used. These simulations, developed by midwives and family nurse‐practitioners, could be used by a variety of learners. Integration of simulations focused on women's and gender‐related health into the training of other health care specialists supports academic preparation for providing person‐centered and culturally responsive care in real‐world clinical settings.^7^, ^28^ Moreover, sharing simulation resources across specialties can be cost‐effective and time‐efficient, maximizing the impact of education investments.

## CONCLUSION

The use of generative AI in simulations allowed midwifery students to practice providing person‐centered telehealth to diverse patient populations. This approach offers several advantages over traditional simulation methods, including exposure to a wider range of patient scenarios, the potential for asynchronous and remote learning, and cost‐effectiveness over multiple cohorts. Although there are limitations, such as ensuring cultural authenticity and measuring long‐term impact, the use of generative AI in midwifery education shows promise for providing safe learning environments and improving the preparation of future midwives to address the unique needs of rural and underserved communities.

## CONFLICT OF INTEREST

The authors have no conflicts of interest to disclose.

## Supporting information




**Appendix S1**:Telehealth Simulations with Generative Artificial Intelligence in Midwifery Education: Practice for Person‐Centered and Culturally Responsive Care C‐ Sample Rubric
