# Supplementary material for: Telehealth Simulations with Generative Artificial Intelligence in Midwifery Education: Practice for Person‐Centered and Culturally Responsive Care
Source: J Midwifery Womens Health. 2025 Aug 20;70(6):932–8. doi: 10.1111/jmwh.70015 (PMC12716106; doi:10.1111/jmwh.70015)
Supplement: Supplementary file 1 — Appendix S1:Telehealth Simulations with Generative Artificial Intelligence in Midwifery Education: Practice for Person‐Centered and Culturally Responsive Care C‐ Sample Rubric [file JMWH-70-932-s001.docx]

**Telehealth Simulations with Generative Artificial Intelligence in Midwifery Education: Practice for Person-Centered and Culturally Responsive Care**

**Rubric 1: Amber**

| **Required* or Optional** | **Item** | **Included** | **Comments** |
| --- | --- | --- | --- |
| **Activity 1** | | | |
| Needed Topics to Cover in the Visit | | | |
|  | Social introduction |  |  |
| Required | Student introduces themself by name and including role on the healthcare team |  |  |
| Required | Preferred language |  |  |
| Required | Name they go by |  |  |
| Required | Pronouns |  |  |
| Required | Menstrual History |  |  |
| Required | Still using IUD? |  |  |
| Required | Location of IUD strings |  |  |
| Optional | Are you having any abdominal pain? |  |  |
| Required | Desire for contraception or pregnancy |  |  |
| Required | Has she taken a pregnancy test? Results? |  |  |
| Required | Obstetric History |  |  |
| Required | Breast-feeding status |  |  |
| Required | Medical History |  |  |
| Required | Surgical History |  |  |
| Required | Current medications and supplements |  |  |
| Required | Current pregnancy symptoms especially pain |  |  |
| Optional | Safety at home / Interpersonal violence |  |  |
| Additional topics related to differentials?  No | | | |

**Rubric 2: Ubah**

| **Required* or Optional** | **Item** | **Included** | **Comments** |
| --- | --- | --- | --- |
| **Visit Planning – Prior to seeing patient** | | | |
| Differential Diagnoses based on Chief Concern -only | | | |
| Required | Pregnancy - normal |  |  |
| Required | Ectopic Pregnancy |  |  |
| Required | Chronic medical conditions affecting pregnancy |  |  |
| Required | Medication use affecting pregnancy |  |  |
| Needed Topics to Cover in the Visit | | | |
|  | Social introduction |  |  |
| Required | Student introduces themself by name and including role on the healthcare team |  |  |
|  | Is English your preferred language for the visit? If not, what language would you like to use? |  |  |
| Required | Name they go by |  |  |
| Required | Pronouns |  |  |
| Required | Desire for current pregnancy |  |  |
| Required | Menstrual History |  |  |
| Required | Obstetric History |  |  |
| Required | Medical History |  |  |
| Required | Surgical History |  |  |
| Required | Current medications and supplements |  |  |
|  | Family Medical History |  |  |
| Required | Current pregnancy symptoms |  |  |
| Required | Safety at home / Interpersonal violence |  |  |
| Optional | Workplace safety – chemicals, lifting |  |  |
| Optional | Housing |  |  |
| Optional | Food |  |  |
| Optional | Adequacy or insecurity |  |  |
| Optional | Unpasteurized cheeses or milk |  |  |
| Optional | Any high-lead fish varieties |  |  |
| Required | Exposure to cat feces |  |  |
| Optional | Other Social/Structural drivers of health |  |  |
| **Assessment** | | | |
| Required | G1P0 woman with a positive pregnancy test |  |  |
| Required | Pregnancy of unknown location |  |  |
| Required | Calculate due date using Naegle’s rule |  |  |
| **Plan** | | | |
|  | Early Pregnancy Counseling |  |  |
| Required | Normal changes in appetite, nausea |  |  |
| Optional | Breast changes |  |  |
| Required | Sexuality |  |  |
| Required | Strategies to avoid toxoplasmosis (do not clean litter box and/or change it every 24 hours, wear gloves while gardening, wash hands with soap after changing the box or gardening) |  |  |
| Required | Do not consume unpasteurized cheeses, milks, or purchased juices |  |  |
| Required | Early Pregnancy Warning Signs |  |  |
|  | Extreme pain – especially one-sided pain in abdomen |  |  |
|  | Chest pain |  |  |
|  | Vaginal bleeding |  |  |
|  | Shortness of breath |  |  |
| Required | Discontinue ibuprofen and use only acetaminophen for headaches |  | Potential increase in gastroschisis and heart defects with use |
| Required | Prescription for folic acid 1,000 mcg OR a prenatal multi-vitamin |  |  |
|  | Lab orders |  |  |
| Required | Hemoglobulin electrophoresis |  |  |
| Required | CBC |  |  |
| Required | Syphilis - VDRL |  |  |
| Required | HIV |  |  |
| Required | Blood Type & Rh |  |  |
| Required | Antibody screen |  |  |
| Required | Rubella Titre |  |  |
| Required | TSH |  |  |
| Optional | Vitamin D |  |  |
| Optional | B12 |  |  |
| Required | Urine |  |  |
| Required | 1^st^ catch – Gonorrhea and Chlamydia |  |  |
| Required | Clean catch – urine culture |  |  |
| Optional | Referral to genetic counseling if patient desires |  |  |
| Optional | Ultrasound after 6 weeks gestation if desired by patient |  |  |
| Required | Come to clinic in 2-4 weeks depending on needs and lab results or if needs a proof of pregnancy test |  |  |

**Format reflective of the Simulation Design Template (revised May 2019) © 2019, National League for Nursing**
